# Accumulation Pattern of Flavonoids during Fruit Development of *Lonicera maackii* Determined by Metabolomics

**DOI:** 10.3390/molecules26226913

**Published:** 2021-11-16

**Authors:** Zengxing Qi, Ran Zhao, Jing Xu, Yanrui Ge, Ruofan Li, Ruili Li

**Affiliations:** 1 National Engineering Laboratory for Tree Breeding, College of Biological Sciences and Technology, Beijing Forestry University, No. 35, Qinghua East Road, Beijing 100083, China; qizengxing@bjfu.edu.cn (Z.Q.); zhaoran@bjfu.edu.cn (R.Z.); xujing@bjfu.edu.cn (J.X.); geyanrui@bjfu.edu.cn (Y.G.); liruofan@bjfu.edu.cn (R.L.); 2 Key Laboratory of Genetics and Breeding in Forest Trees and Ornamental Plants, Ministry of Education, College of Biological Sciences and Technology, Beijing Forestry University, No. 35, Qinghua East Road, Beijing 100083, China; 3 The Tree and Ornamental Plant Breeding and Biotechnology Laboratory of National Forestry and Grassland Administration, College of Biological Sciences and Technology, Beijing Forestry University, No. 35, Qinghua East Road, Beijing 100083, China

**Keywords:** *Lonicera maackii*, flavonoids, UPLC-MS/MS, targeted metabolomics analysis

## Abstract

*Lonicera maackii* (Caprifoliaceae) is a large, upright shrub with fruits that contain many bioactive compounds. Flavonoids are common active substances in *L. maackii*. However, there is a dearth of information about the accumulation of these flavonoids and their possible medicinal value. We used targeted metabolomics analysis based on ultra-performance liquid chromatography-tandem mass spectrometry (UPLC-MS/MS) to analyze five developmental stages of *L. maackii* fruit. A total of 438 metabolites were identified in the five developmental stages, including 81 flavonoids and derivatives. The 81 flavonoids included 25 flavones and derivatives, 35 flavonols and derivatives, two isoflavones, three cyanidins and derivatives, eight procyanidins, and eight flavanones. In addition, we outlined the putative flavonoid biosynthesis pathway and screened their upstream metabolites. More importantly, we analyzed the accumulation patterns of several typical flavones and flavonols. The results reported here improved our understanding of the dynamic changes in flavonoids during fruit development and contributed to making full use of the medicinal value of *L. maackii* fruit.

## 1. Introduction

*Lonicera maackii* (Rupr.) Maxim. (Caprifoliaceae) (common name = Amur honeysuckle) contains many bioactive compounds with potential health-related properties. These include phenolics (flavonoids), chlorogenic acids and derivatives [1,2,3]. *Lonicera maackii* is a large upright shrub cultivated in many northeast Asian countries. It has invaded and become established in the central and northeastern USA [4,5]. Many studies have reported the ornamental value of *L. maackii* [6,7]. *L. maackii* extracts also appear to have medicinal potential such as antioxidant activity [8], liver protection [9], anti-tumor activity [10], and hypoglycemic effects [9]. Its medical potential may be associated with the high levels of rutinoside, luteolin, chlorogenic acid and iridoids [2,3].

Flavonoids have a core structure of 2-phenylchromone. They are a large family of phenolic secondary metabolites widely distributed in flowering plants [11]. They mainly occur in the fruits, roots, stems, leaves, and flowers of plants as complex mixtures of different components, including flavones, flavonols, flavanones, cyanidins, and isoflavones [12,13]. Flavonoids have many pharmacological activities in humans, including anti-tumor, anti-leukemic, anti-cardiovascular disease, anti-inflammatory, anti-oxidative, liver protection and detoxification, and immunomodulatory [14,15,16,17,18,19]. The two most common flavonoid components are quercetin and rutin [20]. Moreover, they have many biological functions during plant growth, development and environmental adaptation. These functions include providing pigments to fruits, flowers, and seeds to attract animals and pollinators for seed dispersal [21], participating in resistance responses [22], improving the germination of pollen [23] and UV protection [24].

Metabolomics uses metabolic information to reveal endogenous metabolic changes in plant systems [25,26]. A variety of tools are used for metabolomics analysis including high-performance liquid chromatography (HPLC) [27], liquid chromatography-tandem mass spectrometry (LC-MS) [28], gas chromatography-tandem mass spectrometry (GC-MS) [29], mass spectrometry (MSn) [30], thin layer chromatography (TLC)–UV spectrophotometry [31], capillary electrophoresis (CE) [32], electrochemistry [33], and NMR-spectrometry [34]. Currently, ultra-high performance liquid chromatography-mass spectrometry (UPLC-MS/MS) is a leading technology for detecting plant-based metabolites and is often used as a guide for the separation and identification of phytochemicals [35]. Flavonoids have been identified by UPLC-MS/MS in plants such as *Dalbergia odorifera* [36], *Crescentia cujete* [37], and *Castanea mollissima* [38]. However, metabolomics has not yet been used to study the flavonoids present during *L. maackii* fruit development.

Therefore, it is important to determine the accumulation pattern of metabolites to control fruit quality and to identify the best collection time of *L. maackii* fruit for medicinal evaluation. In this study, we used a UPLC-MS/MS-based metabolomic analysis approach to study five different developmental stages of *L. maackii* fruit. We studied *L. maackii* metabolomics and variations of flavonoids during fruit development. We also outlined the putative flavonoid biosynthesis pathway. We identified 14 related intermediate metabolites and arranged them according to their corresponding positions in the flavonoid biosynthesis pathway. These metabolome data provide a foundation for the use and development of *L. maackii* chemicals. The results also provide information useful for the extraction of flavonoids and identification of the genes involved in flavonoid biosynthesis.

## 2. Results and Discussion

### 2.1. Overview of Metabolomics Analysis during L. maackii Fruit Development

**Figure 1:**
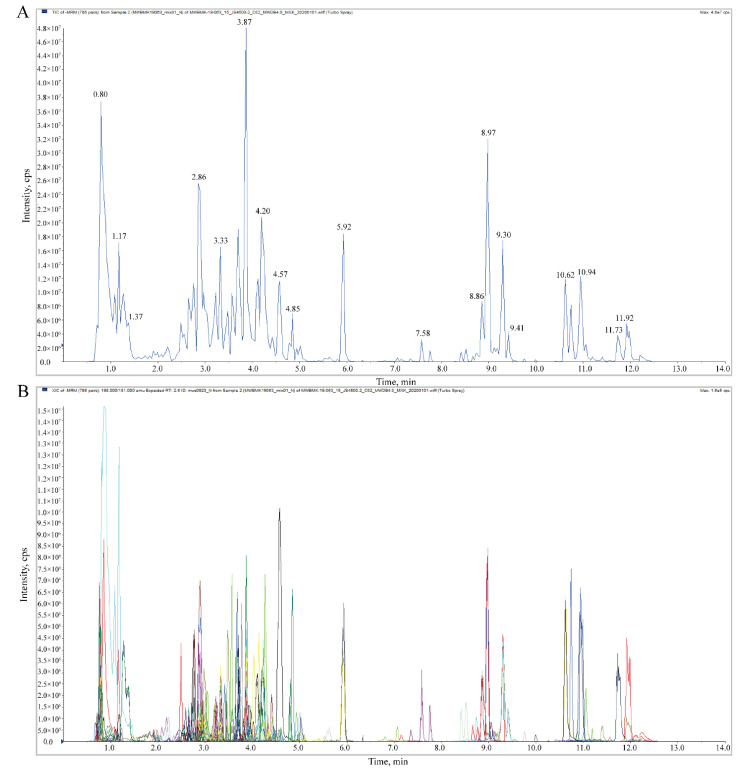
TIC of mixed QC samples by mass spectrometry detection (**A**) and multi-peak detection plot of metabolites in the MRM mode (**B**). The abscissa represents the retention time (RT) of the metabolites and the ordinate represents the current intensity of ion detection.

To study metabolite changes during fruit development, the accumulation pattern of metabolites in fruit was determined by metabolome analysis. The total ions current (TIC) diagram represents a continuous graph obtained by adding the intensity of all ions in the mass spectrum at each time point. Figure 1A shows a typical TIC plot of mixed sample mass spectrometry analysis. Figure 1B shows the multi-peak map of metabolite detection in the multi-reaction monitoring model (MRM). This result showed the substances that can be detected in the sample, with each mass spectrum peak of different colors representing a detected metabolite. The repeatability of metabolite detection could be judged by performing overlapping display and analysis on the TIC diagrams of different QC samples with mass spectrometry detection and analysis (see Supplementary Material Figure S1). The curves of the TIC overlapping diagram have a high degree of overlap, indicating that the retention time and peak intensity are consistent.

**Figure 2:**
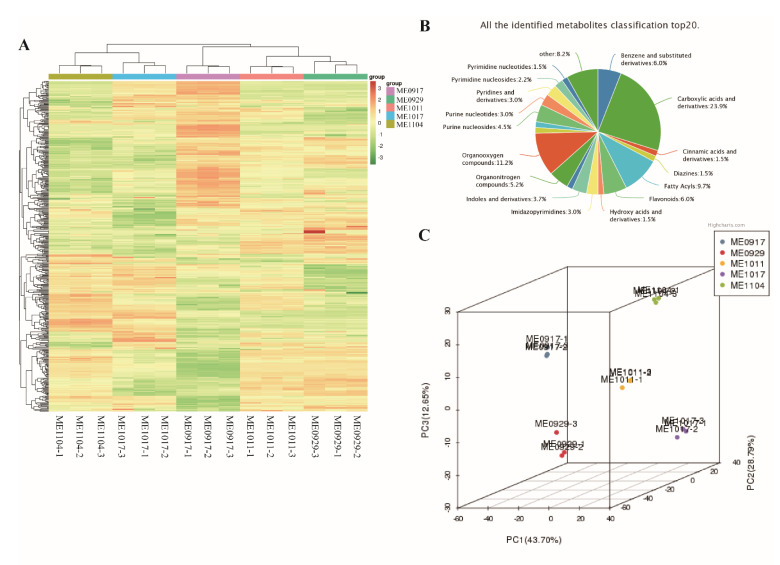
Qualitative and quantitative analysis of metabolome data in five developmental stages of *L. maackii* fruit. (**A**) Clustering heatmap analysis of all metabolites. Each column represents an independent replicate of each stage. Each row represents a different metabolite. The color scale from green (low) to red (high) represents the content of metabolites. (**B**) The top 20 classifications of all identified metabolites. (**C**) Principal component analysis 3D view of five stages of fruit development.

Using UPLC-MS/MS analysis, a total of 438 metabolites were detected in 15 samples of *L. maackii* fruit at five development stages (each stage with three biological replicates) (Table S1). They group into 32 carboxylic acids and derivatives, 15 organooxygen compounds, 13 fatty acids and eight kinds of benzene and substituted derivatives (Figure 2B, Table S2). The accumulation pattern of metabolites among *L. maackii* fruit samples can be visualized through a hierarchical clustering heatmap analysis (Figure 2A). Cluster analysis showed that the biological repeats of all the samples were clustered together (top side of the figure), which suggests the high reliability of the metabolome data. Figure 2A shows that there was obvious separation among the samples in the five periods. In this study, PC1 and PC2 were extracted, which were 37.97% and 28.84%, respectively. The cumulative contribution rate reached 66.81%. The principal component analysis (PCA) score plot showed that M1 (ME0917), M2 (ME0929), M3 (ME1011), M4 (ME1017), M5 (ME1104), and mix (the QC samples mentioned above) were obviously separated, and the repeated samples were compactly collected together (Figure S2). The mixture, including QC samples, was close to the center of the PCA. The results indicated the reliability and reproducibility of the experiment. The PCA 3D view also showed significant separations between ME0917, ME0929, ME1011, ME1017 and ME1104 (Figure 2C). The three biological replicates of each stage had similar PC scores, indicating that the characteristics of the metabolites in the five stages of fruit were significantly distinct, but the replicates were homogeneous. The five developmental stages had distinct metabolite profiles.

### 2.2. Analysis of Differential Metabolites during Fruit Development

**Figure 3:**
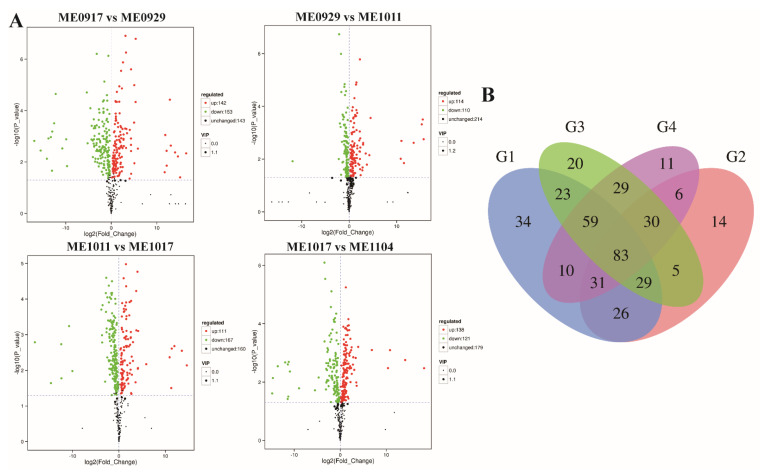
Differential accumulation of metabolites in different developmental stages of *L. maackii* fruit. (**A**) DAMs Volcano map. A number of differential metabolites and expression of up-/downregulation among ME0917 vs. ME0929, ME0929 vs. ME1011, ME1011 vs. ME1017, ME1017 vs. ME1104. (**B**) Venn diagram analysis of DAMs in the four comparison groups. (G1) ME0917 vs. ME0929 (G2) ME0929 vs. ME1011 (G3) ME1011 vs. ME1017 (G4) ME1017 vs. ME1104.

**Figure 4:**
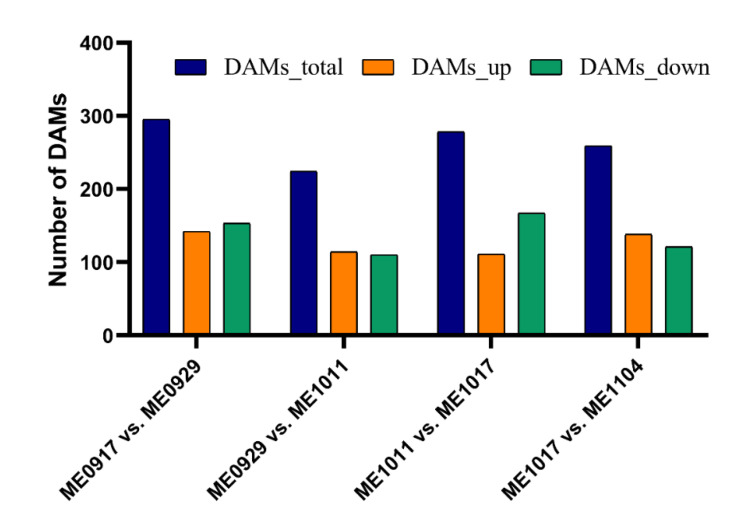
Statistics of the number of DAMs in four pairwise comparisons.

To investigate metabolite changes among different stages of fruit development, the differentially accumulated metabolites (DAMs) were analyzed among ME0917 vs. ME0929, ME0929 vs. ME1011, ME1011 vs. ME1017, and ME1017 vs. ME1104 (Figure 3). Choosing adjacent periods for comparison could more intuitively observe the changes of metabolites over time and find the differential metabolites. The differentially accumulated metabolites between a pair of samples were ascertained with the criteria of FC (fold change) >1 or <1, *p*-value < 0.05 and VIP > 1, and the results were visualized using a volcano plot (Figure 3A). Many differential metabolites were found in the four differential groups. In concrete terms, there were 295 DAMs between ME0917 and ME0929: 153 downregulated and 142 upregulated (Figure 4), including 14 flavones and derivatives, 19 flavonols and derivatives, six flavanones and other compounds (Table S3). A total of 224 metabolites with significant differences were identified between ME0929 and ME1011, of which 114 were upregulated and 110 were downregulated (Figure 4). The 224 DAMs included including 14 flavones and derivatives, 23 flavonols and derivatives, three flavanones and other compounds (Table S4). In ME1011 vs. ME1017, there were 167 downregulated DAMs and 111 upregulated DAMs (Figure 4), including 15 flavones and derivatives,15 flavonols and derivatives, six flavanones and other compounds (Table S5). In total, 138 upregulated DAMs and 121 downregulated DAMs were identified between ME1017 vs. ME1104 (Figure 4), including 15 flavones and derivatives, 23 flavonols and derivatives, four flavanones and other compounds (Table S6). The Venn diagram shows that there were 83 common differential metabolites in the four combinations (Figure 3B and Table S10). These 83 common metabolites included 16 differentially accumulated flavonoids (DAFs) (Table S10). Figure S3 shows the variations of 9 DAFs with high accumulation levels in different growth stages.

### 2.3. KEGG Annotation and Enrichment Analysis of Differential Metabolites

**Figure 5:**
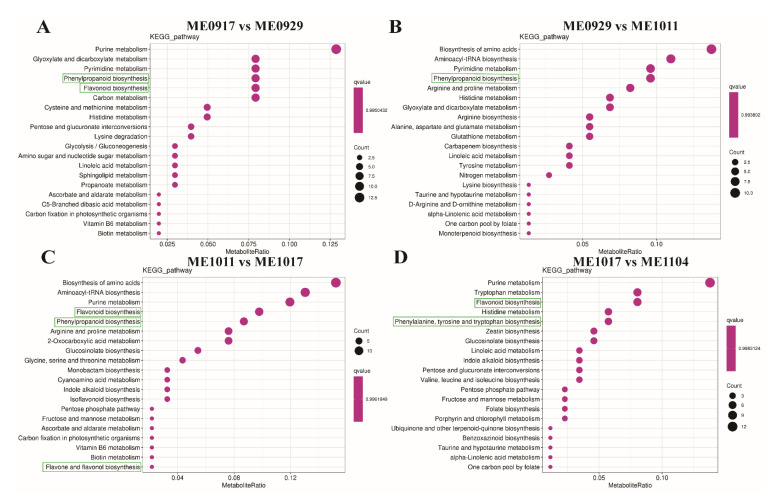
The top 20 KEGG pathways assignment of the DAMs among ME0917 vs. ME0929 (**A**), ME0929 vs. ME1011 (**B**), ME1011 vs. ME1017 (**C**), and ME1017 vs. ME1104 (**D**). The size of the dot represents the count of differentially accumulated metabolites that were enriched in the corresponding pathway.

To explore the biological processes of the DAMs and the potential metabolism mechanisms of flavonoids during fruit development, we used the Kyoto Encyclopedia of Genes and Genomes (KEGG) pathway enrichment analysis to identify metabolic pathways and signal pathways. These differentially accumulated metabolites were matched to 85 pathways. Figure 5 and Table S7 show that the DAMs of ME0917 vs. ME0929 were significantly involved in phenylpropanoid biosynthesis (ko00940), flavonoid biosynthesis (Ko00941), and phenylalanine metabolism (ko00360) (Figure 5A). The DAMs of ME0929 vs. ME1011 were mainly enriched in phenylpropanoid biosynthesis (ko00940) (Figure 5B). Additionally, different metabolites of ME1011 vs. ME1017 were assigned to flavonoid biosynthesis (ko00941), phenylpropanoid biosynthesis (ko00940), and flavone and flavonol biosynthesis (ko00944) (Figure 5C). For ME1017 vs. ME1104, differentially accumulated metabolites were designated to flavonoid biosynthesis (ko00941), phenylalanine, tyrosine and tryptophan biosynthesis (ko00400) (Figure 5D). The top enriched KEGG terms among the DAMs detected for all the comparison groups were the biosynthesis of secondary metabolites (ko01110), purine metabolism (ko00230) and ABC transporters (ko02010). Although the enriched metabolic pathways were not exactly the same in the four comparison groups, the “phenylpropanoid biosynthesis” and “flavonoid biosynthesis” pathways were significantly enriched in all comparison groups (Table S7). In conclusion, KEGG pathway analysis showed that the dynamic changes of flavonoid accumulation might be due to DAMs involved in these significant enrichment metabolic pathways.

### 2.4. Accumulation Profiles of Flavonoids during Fruit Development

Flavonoids are widely used in food, medicine and health care owing to their broad spectrum of pharmacological activities [39,40]. They also function in plant disease resistance and immunity [41]. Few studies have qualitatively and quantitatively studied the flavonoid metabolites in the fruit of *L. maackii*. In this study, 81 flavonoids and derivatives were identified in developing *L. maackii* fruit (Table S8), including 25 flavones and derivatives (Table 1), 35 flavonols and derivatives (Table 1), two isoflavones (Table S8), three cyanidins and derivatives (Table S8), eight procyanidins (Table S8), and eight flavanones (Table S8).

**Figure 6:**
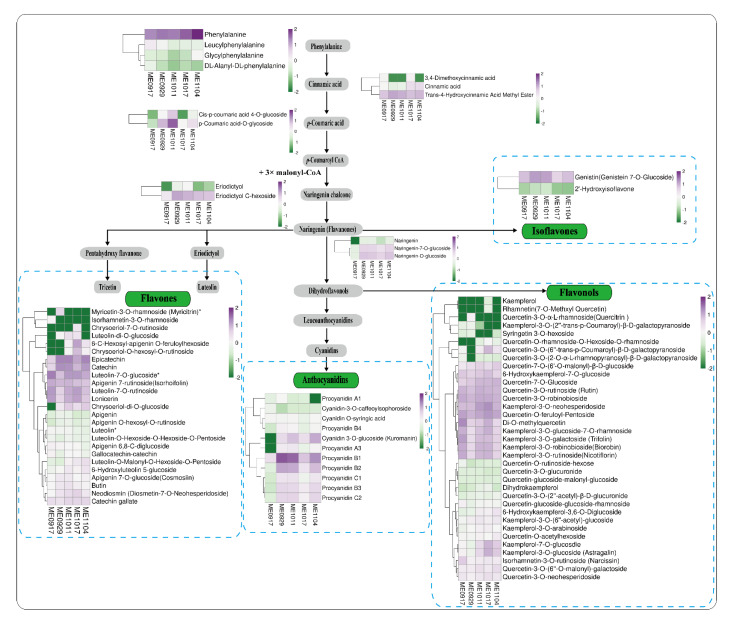
Reconstruction of flavonoid biosynthesis pathway and variations of flavonoids contents in *L. maackii* fruit. The key intermediate metabolites of each step of the biosynthesis pathway are shown in the grey box, and flavonoids are shown in the green box. The color scale from green to purple represents the measured accumulation level of metabolites from low to high during fruit development. (Metabolites with * are isomers) (*n* = 3).

To study flavonoid variations during *L. maackii* fruit development, we reconstructed the putative flavonoid biosynthesis pathway based on the KEGG map (Figures S4–S6) and the reported biosynthesis pathway of flavonoids [42]. We identified 14 related intermediate metabolites and rearranged them according to their corresponding positions in the biosynthesis pathway (Figure 6 and Table S9). These metabolites are involved in the phenylpropanoid metabolism pathway and are also involved in the flavonoid biosynthesis pathway.

We analyzed the flavones in *L. maackii* fruit. A total of 25 flavones and derivatives were identified (Figure 6). Metabolite accumulation profile analysis showed that the contents of epicatechin, catechin, luteolin-7-O-glucoside, apigenin 7-rutinoside, luteolin-7-O-rutinoside, and lonicerin were significantly higher than those of other flavones. Catechins, which are multifunctional polyphenols, help reduce reactive oxygen species and improve the environmental adaptability of plants. They also have beneficial health effects such as improving cardiac function, anti-inflammatory, anti-aging, and lipid reduction [43]. The contents of epicatechin and catechin in ME0929, ME1011 and ME1104 were significantly higher than those in ME0917 and ME1017. The fold change of epicatechin was significantly increased (4.34-fold) in ME0917 vs. ME0929 (Table 1). The content variations of luteolin-7-O-glucoside, apigenin 7-rutinoside, luteolin-7-O-rutinoside and lonicerin showed opposite trends. The contents of luteolin and apigenin showed no significant difference in the pair-wise comparison, except for ME1011 vs. ME1017. Luteolin mostly exists in plants in the form of glycosides. Luteolin has many pharmacological activities, including being anti-allergic and lowering uric acid levels [44].

We analyzed the flavonols in the *L. maackii* fruit and identified 35 flavonols and derivatives in *L. maackii* fruit (Figure 6). Flavonols were the most diverse flavonoid metabolites detected. Flavonols had the maximum number of flavonoid metabolites with significant differences in accumulation in the five development stages of *L. maackii* fruit. These results showed that the di-O-methylquercetin, quercetin-O-feruloyl-pentoside, kaempferol-3-O-neohesperidoside, kaempferol-3-O-neohesperidoside, and quercetin-O-feruloyl-pentoside were the most abundant flavonols in the five development stages of fruit, respectively. Unfortunately, the accumulation of kaempferol was low and only slightly increased in ME1017. The level of dihydrokaempferol was significantly increased in the pairwise comparison, except for ME1011 vs. ME1017. Kaempferol has many uses, such as the treatment of Parkinson’s disease, and repairing liver damage [45,46]. The more abundant flavonols were mainly concentrated in the three stages of ME0917, ME1011, and ME1017, while the contents of ME0929 and ME1104 were lower, which was similar to the variation trends of luteolin-7-O-glucoside, apigenin 7-rutinoside, luteolin-7-O-rutinoside and lonicerin. The abundant flavonols included di-O-methylquercetin, kaempferol-3-O-glucoside-7-O-rhamnoside, kaempferol-3-O-galactoside, kaempferol-3-O-glucoside, kaempferol-7-O-glucosdie, kaempferol-3-O-robinobioside, kaempferol-3-O-rutinoside. The fold changes of quercetin-7-O-glucoside were increased by 1.01-fold and 0.16-fold in ME0929 vs. ME1011 and ME1017 vs. ME1104, respectively. Both quercetin-3-O-robinobioside and quercetin-3-O-rutinoside exhibited the same trend of an initial decline in ME0917 vs. ME0929, and then an increase in ME0929 vs. ME1011. Quercetin is a food-borne flavonoid with a variety of physiological functions. Quercetin has a therapeutic effect on lipid accumulation and inflammation in non-alcoholic fatty liver and it can reduce the damage caused by a non-alcoholic fatty liver [47]. Rutin (quercetin-3-O-rutinoside) has been studied as a natural flavonoid with biological functions in several pathological situations. Rutin can attenuate neuroinflammation, improve memory deficits and delay the pathological process of Alzheimer’s disease [48].

Previous research has shown that the content of precursor metabolites related to lignin synthesis is significantly negatively correlated with the content of precursor metabolites related to flavonoids biosynthesis [49,50]. Hence ferulic acid and trans-ferulic acid were searched from metabolome data (Table S9). The accumulation levels of trans-ferulic acid and ferulic acid were low in ME0929 and ME1011, while the contents in ME0917 and ME1017 were moderately high. The content change trend of trans-ferulic acid and ferulic acid was opposite that of upstream metabolites of flavonoid biosynthesis, such as eriodictyol C-hexoside, naringenin-7-O-glucoside, naringenin-O-glucoside. Nevertheless, lignin was not detected in any of the samples tested.

Previous studies have not reported the metabolomics of *L. maackii* fruits. The present study is the first description of their metabolite profile. These metabolites have potential medicinal value. Metabolomics analysis of their changes can help identify periods with the greatest levels of targeted metabolites. This information targets the optimal harvest time for fruit containing compounds with medicinal value.

## 3. Materials and Methods

### 3.1. Plant Materials

The fruiting period of *L. maackii* is from August to November. During the development of *L. maackii*, the color of the fruit gradually changed from green to half red and half green, and then completely changed to dark red. Therefore, on 17 September 2019, 29 September 2019, 11 October 2019, 17 October 2019 and 4 November 2019, we collected the *L. maackii* fruits at the Beijing Forestry University (40.008° N, 116.345° E), Beijing, China. All samples (at least 15 fruits per sample) were immediately frozen in liquid nitrogen and stored at −80 °C for subsequent analysis. Widely targeted metabolomics analysis was performed on the five periods of fruit (ME0917, ME0929, ME1011, ME1017, and ME1104). There were three biological replicates (15 samples in total). The samples were identified by Professor Huihong Guo at Beijing Forestry University. We deposited a voucher specimen in the Museum of Beijing Forestry University. The herbarium access code of the voucher is BJFC00123698.

### 3.2. Sample Extraction and UPLC–MS/MS System-Based Metabolomics Analysis

#### 3.2.1. Sample Preparation and Extraction

Biological samples were placed in a freeze dryer (Scientz-100F) for vacuum freeze-drying. The freeze-dried fruit was crushed using a mixer mill (MM 400, Retsch, Dusseldorf, Germany) with zirconia beads for 1.5 min at 30 Hz. A 100-mg sample of powder was weighted and extracted overnight at 4 °C with 0.6 mL 70% aqueous methanol (GR: Guaranteed reagent, Merck, Darmstadt, Germany). Following centrifugation at 10,000× *g* for 10 min, the extracts were absorbed (CNWBOND Carbon-GCB SPE Cartridge, 250 mg, 3 mL; ANPEL, Shanghai, China, www.anpel.com.cn/cnw, accessed on January 2020) and filtrated (SCAA-104, 0.22-μm pore size; ANPEL, Shanghai, China, http://www.anpel.com.cn/, accessed on January 2020). The samples were stored in an injection flask for UPLC-MS/MS analysis. Quality control (QC) samples were prepared using mixed *L. maackii* fruit sample extracts to monitor the reproducibility of the samples under the same treatment method. One QC sample was inserted in each of the 10 detected samples during stability evaluation of the instrumental analysis conditions.

#### 3.2.2. Acquisition Conditions of Chromatography-Mass Spectrometry

The data acquisition instrument system included an ultra-performance liquid chromatography (UPLC) (Shim-pack UFLC SHIMADZU CBM30A, https://www.shimadzu.com.cn/, accessed on January 2020) and a tandem mass spectrometry (MS/MS) (Applied Biosystems 4500 QTRAP, http://www.appliedbiosystems.com.cn/, accessed on January 2020).

UPLC Conditions: The sample extracts were analyzed using an UPLC-ESI-MS/MS system (UPLC, Shim-pack UFLC SHIMADZU CBM30Asystem, www.shimadzu.com.cn/, accessed on January 2020; MS, Applied Biosystems 4500 Q TRAP, www.appliedbiosystems.com.cn/, accessed on January 2020). The analytical conditions were as follows, UPLC: column, Waters ACQUITY UPLC HSS T3 C18 (1.8 µm, 2.1 mm × 100 mm); The mobile phase consisted of solvent A that was pure water with 0.04% acetic acid, and solvent B that was acetonitrile (GR: Guaranteed reagent, Merck) with 0.04% acetic acid. Sample measurements were performed with a gradient program that employed the starting conditions of 95% A, 5% B. Within 10 min, a linear gradient to 5% A, 95% B was programmed, and a composition of 5% A, 95% B was kept for 1 min. Subsequently, a composition of 95% A, 5.0% B was adjusted within 0.10 min and kept for 2.9 min. The column oven was set to 40 °C. The injection volume was 4 μL. The effluent was alternatively connected to an ESI-triple quadrupole-linear ion trap (QTRAP)-MS.

ESI-Q TRAP-MS/MS: LIT and triple quadrupole (QQQ) scans were acquired on a triple quadrupole-linear ion trap mass spectrometer (Q TRAP, Applied Biosystems API 4500 Q TRAP UPLC/MS/MS System, Foster City, CA, USA) equipped with an ESI Turbo Ion-Spray interface operating in positive and negative ion mode and controlled by Analyst 1.6.3 software (AB Sciex, Framingham, MA, USA). The ESI source operation parameters were as follows: ion source, turbo spray; source temperature 550 °C; ion spray voltage (IS) 5500 V (positive ion mode)/−4500 V (negative ion mode); ion source gas I (GSI), gas II (GSII), and curtain gas (CUR) were set at 50, 60, and 30.0 psi, respectively; the collision gas (CAD) was high. Instrument tuning and mass calibration were performed with 10 and 100 μmol/L polypropylene glycol solutions in QQQ and LIT modes, respectively. QQQ scans were acquired as MRM experiments with collision gas (nitrogen) set to 5 psi. DP and CE for individual MRM transitions were done with further DP and CE optimization [51]. A specific set of MRM transitions were monitored for each period according to the metabolites eluted within the period.

### 3.3. Metabolomics Data Analysis

Multivariate statistical analysis methods were used to analyse the metabolome data, including qualitative and quantitative analysis, principal component analysis (PCA), and hierarchical clustering analysis (HCA). Then, the orthogonal partial least squares-discriminant analysis (OPLS-DA) was performed, and the variable importance in projection (VIP) of OPLS-DA model was calculated for the screening of differential metabolites. Differentially accumulated metabolites (DAMs) were screened based on the thresholds [VIP > 1, FC > 1 or <1 and *p*-value of *t*-test < 0.05]. The R (3.3.2) package ropls was used to evaluate the validity of OPLS-DA model. The prediction parameters of the evaluation model include R^2^X, R^2^Y and Q^2^, where Q^2^ represents the predictive ability of the model, and R^2^X and R^2^Y represent the interpretation rates of the built model to X and Y matrices, respectively. The closer these three indicators are to 1, the more stable and reliable the model is. When Q^2^ > 0.5, it can be considered as an effective model, and when Q^2^ > 0.9, it can be considered as an excellent model. Then, the KEGG database was used to annotate and enrich differentially accumulated metabolites [52].

#### 3.3.1. LC-MS Processing Data

Qualitative and quantitative mass spectrometry analysis of metabolites were based on KEGG compound database, self-built database and multiple reaction monitoring (MRM). The interference from isotope signals, repetitive signals of K^+^, Na^+^, and NH4^+^ ions and fragment ions derived from other larger molecules were removed when qualitative analysis of the metabolites was performed.

Metabolite quantification was accomplished with data acquired in the multiple reaction monitoring (MRM) mode of QQQ mass spectrometry. In MRM mode, the quadrupole first screened for precursor ions of target substances while screening out any ions derived from substances of different molecular weights to preliminarily eliminate their interference. The precursor ions were fragmented by induced ionization in the collision chamber to form several fragment ions. Then, unique fragment ions were selected with desired characteristics QQQ and to exclude non-target ions. This step aimed to make the quantification more accurate and improve repeatability. After metabolite mass spectrometry data were obtained for different samples, all mass spectrum peaks were subjected to area integration. The mass spectrum peaks of the same metabolite in different samples were integral-corrected [53]. Metabolite identification was based on the accurate mass of metabolites, MS2 fragments, MS2 fragments, isotope distribution and retention time (RT). Through the self-developed intelligent secondary spectrum matching method, the secondary spectrum and RT of the metabolites in the project samples were compared with the secondary database spectrum and RT were intelligently matched one by one, and the MS tolerance and MS2 tolerance were set to 2 ppm and 5 ppm, respectively.

The mass spectral data were analyzed using Analyst 1.6.3. Based on the local metabolic database, the metabolites of the samples were qualitatively and quantitatively analyzed by mass spectrometry. The characteristic ions of each substance were screened through the triple quadrupole (QQQ), and the signal strength (CPS) of the characteristic ions was obtained in the detector. MultiaQuant software was used to open the sample mass spectrometry file and the integration and calibration of chromatographic peaks were carried out. The peak area (Area) of each chromatographic peak represented the relative content of the corresponding substance. Finally, all chromatographic peak area integral data were derived for storage.

#### 3.3.2. Principal Component Analysis

Principal component analysis (PCA) was performed on all samples, including quality control samples (QC) to preliminarily elucidate the total metabolic differences among the samples of each group and the degree of variation among the samples within the group. Prcomp function of R (http://www.r-project.org/, accessed on February 2020) was used for PCA. Set the prcomp function parameter scale = True, indicating that the data was normalized by unit variance scaling (UV). PCA results showed the tendency of metabolome separation among each group, indicating differences in the metabolome between sample groups.

#### 3.3.3. Clustering Analysis

Metabolites content data were normalized by unit variance scaling (UV), and heatmaps were drawn by the R software pheatmap package (www.r-project.org/, accessed on February 2020). The accumulation patterns of metabolites among different samples were analyzed by hierarchical clustering analysis (HCA).

## 4. Conclusions

Metabolomics analysis revealed differentially accumulated metabolites during *L. maackii* fruit growth. A total of 438 metabolites were identified, including 81 flavonoids and their derivatives. The 81 flavonoids included 25 flavones and derivatives, 35 flavonols and derivatives, two isoflavones, three cyanidins and derivatives, eight procyanidins, and eight flavanones. KEGG enrichment showed that many differential metabolites were involved in phenylpropanoid biosynthesis, phenylalanine metabolism, flavone and flavonol biosynthesis, flavonoid biosynthesis and biosynthesis of secondary metabolites. Therefore, it is likely that these DAMs are the key metabolites underlying the dynamic variations of flavonoids. The metabolomics analysis highlighted the significant variations in the levels of flavonoids with potential medicinal value. We screened out the upstream metabolites and proposed the flavonoid biosynthesis pathway in the fruit of *L. maackii*. These data increase knowledge of the accumulation pattern of flavonoids and provide a basis for exploring the related genes involved in flavonoids biosynthesis of *L. maackii* fruit.

## Figures and Tables

**Table 1:** Targeted metabolomics study on the accumulation profiles of flavones and flavonols in different development stages of *L. maackii* fruit. (*n* = 3) (T1 = ME0917, T2 = ME0929, T3 = ME1011, T4 = ME1017, T5 = ME1104.) (“Log2FC > 0” represents “upregulation”; “Log2FC < 0” represents “downregulation”; “-” represents “no change”).

Metabolite Name	T1 vs. T2		T2 vs. T3		T3 vs. T4		T4 vs. T5	
**Flavone**	**Log2FC**	**VIP**	**Log2FC**	**VIP**	**Log2FC**	**VIP**	**Log2FC**	**VIP**
Isorhamnetin-3-O-rhamnoside	−12.97	1.14	-	-	-	-	-	-
Myricetin-3-O-rhamnoside	-	-	-	-	-	-	-	-
Luteolin	-	-	-	-	−0.88	1.10	-	-
Luteolin-7-O-glucoside	−3.56	1.12	2.26	1.25	-	-	−1.13	1.14
6-Hydroxyluteolin 5-glucoside	-	-	1.89	1.22	−0.95	1.06	1.42	1.18
Luteolin-di-O-glucoside	-	-	2.13	1.14	-	-	−14.79	1.15
Luteolin-O-Malonyl-O-Hexoside-O-Pentoside	-	-	3.72	1.20	-	-	-	-
Luteolin-O-Hexoside-O-Hexoside-O-Pentoside	−1.57	1.09	1.56	1.22	−0.85	1.06	-	-
Apigenin	-	-	-	-	−2.14	1.17	-	-
Apigenin 7-O-glucoside	1.50	1.13	-	-	−1.48	1.18	0.86	1.09
Apigenin 7-rutinoside	−0.80	1.09	-	-	-	-	-	-
Apigenin 6,8-C-diglucoside	-	-	-	-	-	-	−1.30	1.19
Apigenin O-hexosyl-O-rutinoside	2.30	1.07	-	-	−1.33	1.17	-	-
6-C-Hexosyl-apigenin O-feruloylhexoside	-	-	13.58	1.26	5.90	1.17	−4.69	1.19
Butin	1.83	1.13	0.45	1.18	−1.30	1.18	−0.70	1.18
Chrysoeriol-7-O-rutinoside	-	-	-	-	14.64	1.17	−14.64	1.19
Chrysoeriol-di-O-glucoside	14.65	1.13	2.41	1.17	−1.53	1.04	1.48	1.16
Chrysoeriol-O-hexosyl-O-rutinoside	-	-	-	-	-	-	-	-
Luteolin-7-O-rutinoside	−3.27	1.14	2.30	1.20	0.79	1.09	−2.07	1.19
Diosmetin-7-O-Neohesperidoside	1.95	1.14	1.13	1.24	1.06	1.16	−2.60	1.20
Epicatechin	4.34	1.14	-	-	−1.83	1.18	1.77	1.20
Catechin	4.33	1.14	−0.33	1.10	−2.05	1.17	1.91	1.19
Lonicerin	−3.14	1.14	2.27	1.23	0.72	1.14	−2.34	1.20
Catechin gallate	-	-	1.28	1.19	-	-	-	-
Gallocatechin-catechin	−2.70	1.14	2.26	1.26	-	-	−1.30	1.16
**Flavonol**	**Log2FC**	**VIP**	**Log2FC**	**VIP**	**Log2FC**	**VIP**	**Log2FC**	**VIP**
Kaempferol	-	-	-	-	0.59	1.15	−11.28	1.13
Dihydrokaempferol	0.76	1.06	1.68	1.26	-	-	−2.92	1.18
Kaempferol-3-O-arabinoside	-	-	-	-	0.44	1.14	−1.13	1.20
Kaempferol-7-O-glucosdie	−1.97	1.12	4.51	1.26	2.58	1.18	−1.40	1.20
Kaempferol-3-O-glucoside	−1.66	1.11	4.24	1.25	2.63	1.18	−1.34	1.19
Kaempferol-3-O-galactoside	−3.72	1.14	2.16	1.25	-	-	−1.25	1.17
6-Hydroxykaempferol-7-O-glucoside	−0.36	1.04	-	-	-	-	-	-
Kaempferol-3-O-(6″-acetyl)-glucoside	1.69	1.07	-	-	-	-	0.80	1.11
Kaempferol-3-O-(2″-trans-p-Coumaroyl)-β-d-galactopyranoside	-	-	-	-	-	-	-	-
Kaempferol-3-O-robinobioside	−2.90	1.13	2.33	1.26	-	-	−1.75	1.19
Kaempferol-3-O-rutinoside	−2.81	1.14	2.23	1.26	-	-	−1.80	1.18
Kaempferol-3-O-neohesperidoside	-	-	1.53	1.26	0.71	1.18	−1.91	1.20
Kaempferol-3-O-glucoside-7-O-rhamnoside	−3.20	1.14	2.35	1.25	0.81	1.12	−2.14	1.18
6-Hydroxykaempferol-3,6-O-Diglucoside	-	-	2.82	1.21	-	-	1.19	1.17
7-O-Methxyl Quercetin	-	-	-	-	11.96	1.18	−11.96	1.20
Di-O-methylquercetin	−3.84	1.14	0.33	1.19	1.58	1.18	−2.63	1.20
Quercetin-3-O-α-l-rhamnoside	-	-	-	-	-	-	-	-
Quercetin-7-O-Glucoside	-	-	1.01	1.22	-	-	0.16	1.05
Quercetin-3-O-glucuronide	-	-	-	-	-	-	-	-
Quercetin-O-acetylhexoside	-	-	-	-	-	-	1.42	1.15
Quercetin-3-O-(2″-acetyl)-β-d-glucuronide	-	-	-	-	-	-	0.81	1.11
Quercetin-3-O-(6″-O-malonyl)-galactoside	-	-	1.12	1.25	−0.73	1.18	1.25	1.17
Quercetin-7-O-(6′-O-malonyl)-β-d-glucoside	-	-	1.27	1.16	−1.17	1.17	1.20	1.19
Quercetin-3-O-(6″-trans-p-Coumaroyl)-β-d-galactopyranoside	−13.27	1.14	15.28	1.26	4.16	1.18	−3.40	1.20
Quercetin-O-feruloyl-Pentoside	−0.95	1.14	0.85	1.22	0.61	1.14	−0.80	1.19
Quercetin-3-O-rutinoside (Rutin)	−0.67	1.01	0.80	1.09	-	-	-	-
Quercetin-3-O-robinobioside	−0.72	1.07	0.68	1.19	-	-	-	-
Quercetin-3-O-(2-O-α-l-rhamnopyranosyl)-β-d-galactopyranoside	−13.03	1.10	11.00	1.26	-	-	-	-
Quercetin-3-O-neohesperidoside	−1.04	1.06	0.88	1.13	0.72	1.10	−0.76	1.15
Quercetin-glucoside-malonyl-glucoside	-	-	-	-	-	-	-	-
Quercetin-O-rhamnoside-O-Hexoside-O-rhamnoside	-	-	11.52	1.23	2.63	1.08	−2.04	1.08
Quercetin-O-rutinoside-hexose	−3.62	1.14	0.96	1.17	0.70	1.12	-	-
Quercetin-glucoside-glucoside-rhamnoside	−2.70	1.14	1.14	1.07	-	-	-	-
Syringetin 3-O-hexoside	-	-	−11.92	1.24	-	-	-	-
Isorhamnetin-3-O-rutinoside	−3.25	1.14	-	-	-	-	-	-

## Data Availability

Data is contained within the article or Supplementary Materials.

## Supplementary Materials

The following are available online. Figure S1: The TIC overlay diagram of QC sample mass spectrometry detection, Figure S2: The PCA score plot of each group sample and QC samples, Figure S3: The variation trends of 9 DAFs in the five developmental stages, Figure S4: KEGG map of “phenylpropanoid biosynthesis”, Figure S5: KEGG map of “flavonoid biosynthesis”, Figure S6: KEGG map of “flavone and flavonol biosynthesis”, Table S1: All metabolites were detected in the five developmental stages of *L. maackii* fruit, Table S2: The top 20 classifications of all identified metabolites, Table S3: A list of the DAMs between ME0917 vs. ME0929, Table S4: A list of the DAMs between ME0929 vs. ME1011, Table S5: A list of the DAMs between ME1011 vs. ME1017, Table S6: A list of the DAMs between ME1017 vs. ME1104, Table S7: KEGG pathways enrichment of the DAMs in four comparison groups, Table S8: A list of the 81 flavonoids and derivatives detected in this study, Table S9: A list of the pivotal precursor metabolites in flavonoids and lignin synthesis pathway, Table S10: A list of the common DAMs and common DAFs in the four comparison groups.

## Sample Availability

Samples of the *L. maackii* fruit are available from the authors.
