# Supplementary figures and images for: Accumulation Pattern of Flavonoids during Fruit Development of Lonicera maackii Determined by Metabolomics
Source: Molecules. 2021 Nov 16;26(22):6913. doi: 10.3390/molecules26226913 (PMC8624894; doi:10.3390/molecules26226913)

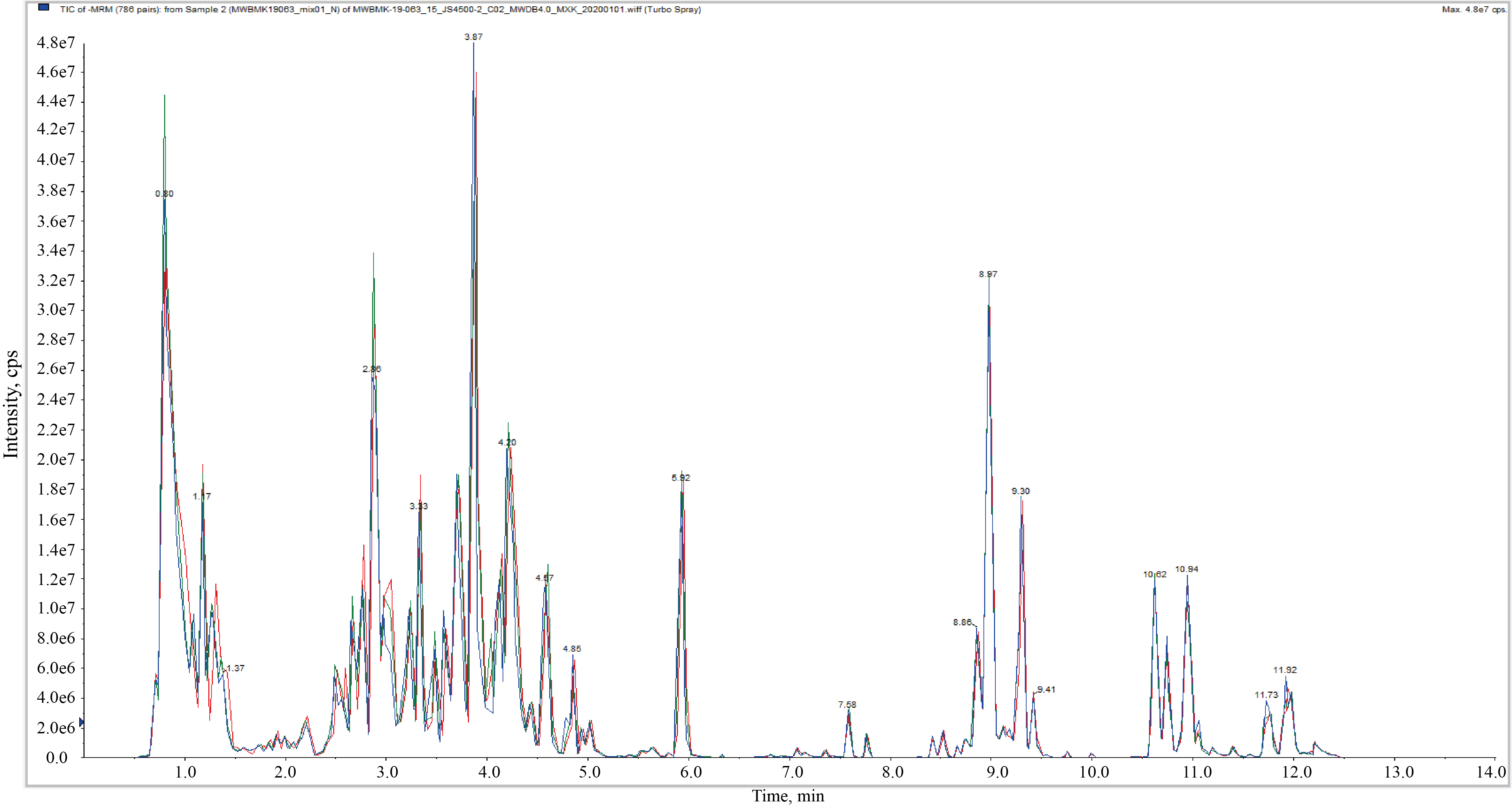

Supplement: Supplementary file 1 [file molecules-26-06913-s001.zip › supplementary materials/Figure S1.png]

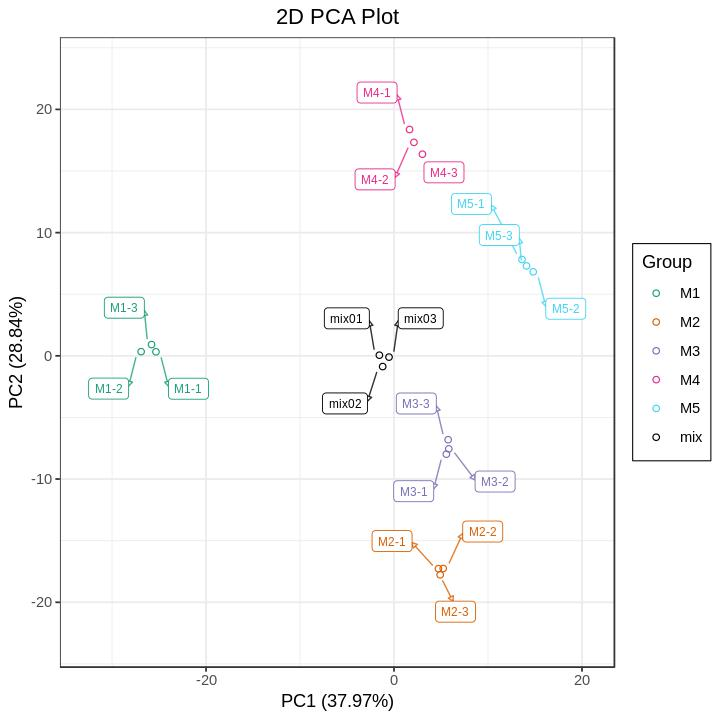

Supplement: Supplementary file 1 [file molecules-26-06913-s001.zip › supplementary materials/Figure S2.png]

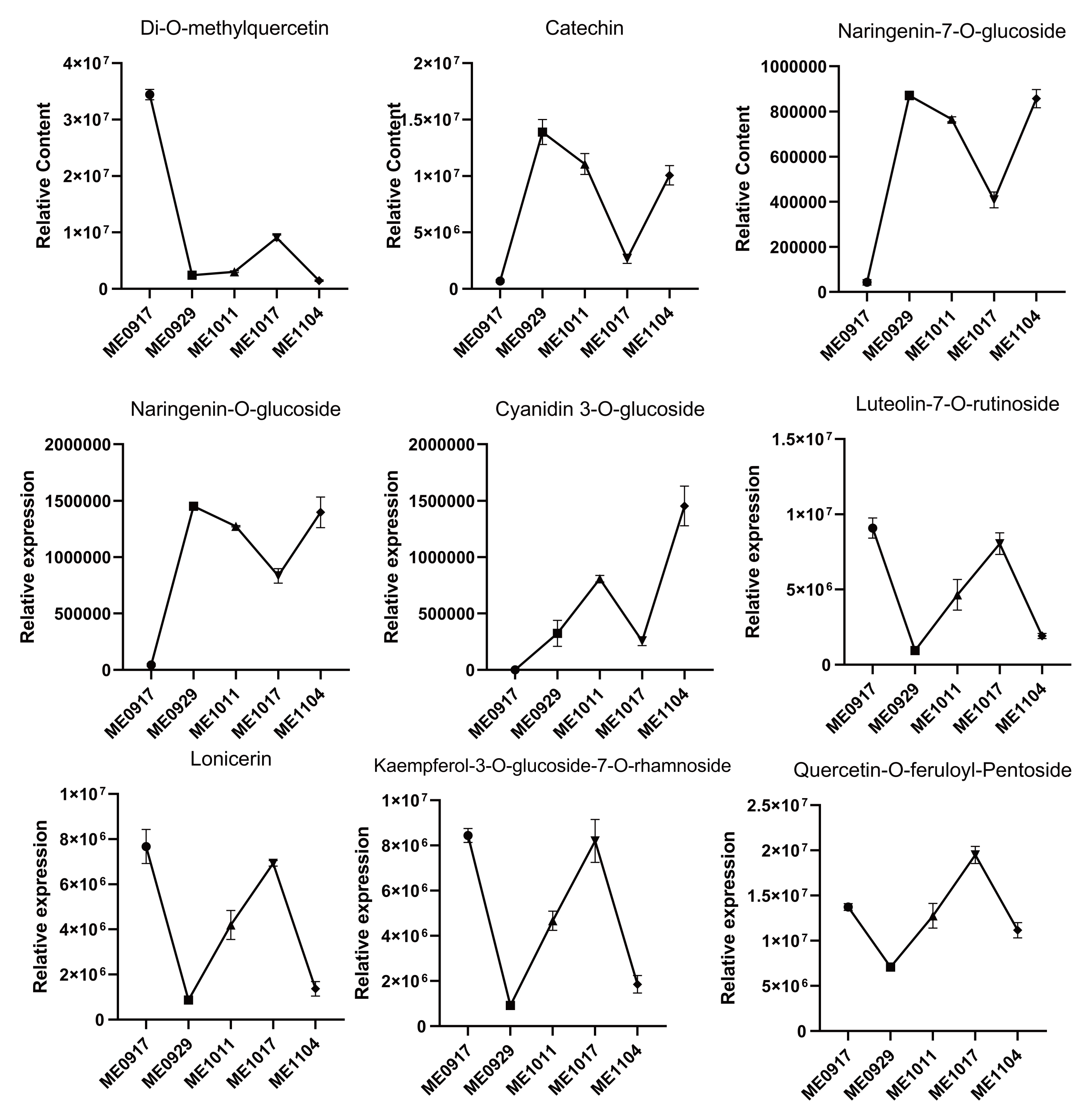

Supplement: Supplementary file 1 [file molecules-26-06913-s001.zip › supplementary materials/Figure S3.png]

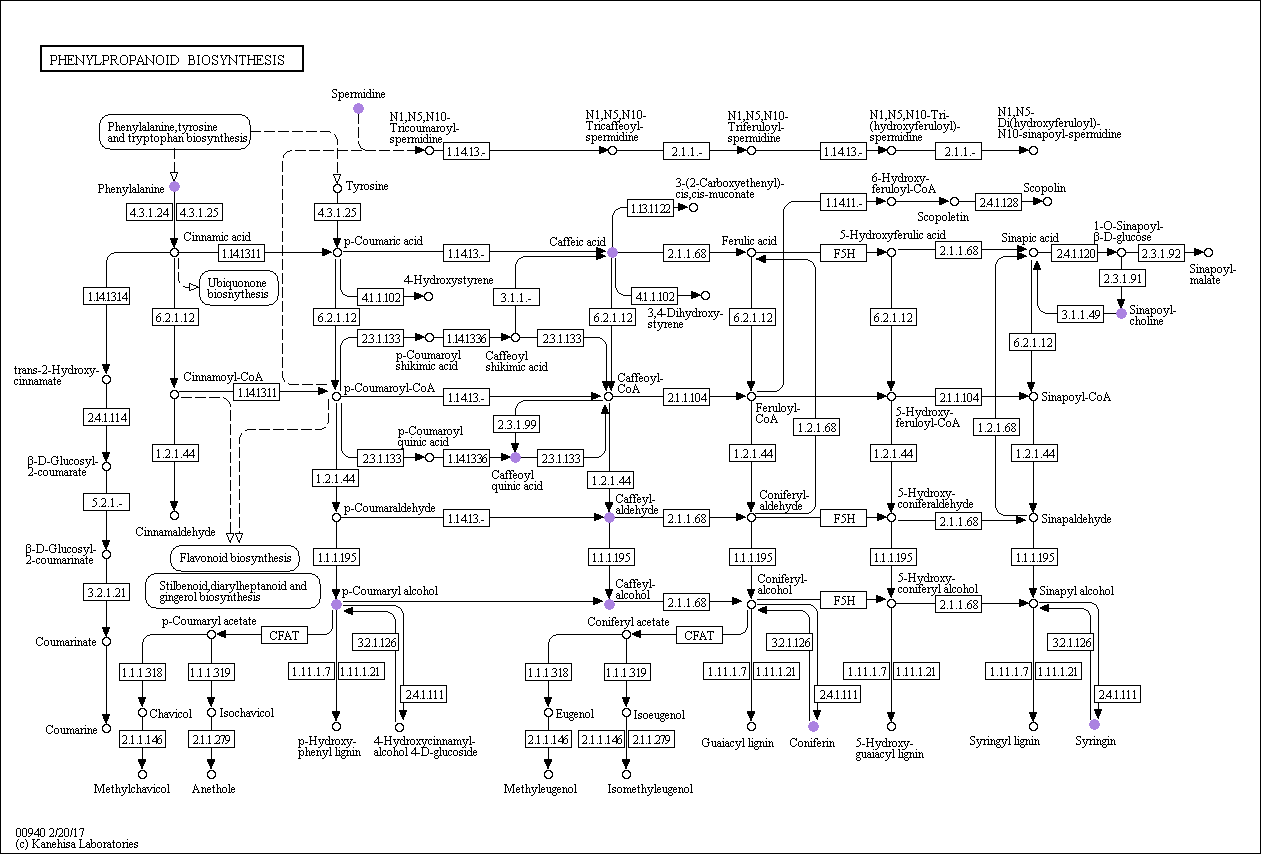

Supplement: Supplementary file 1 [file molecules-26-06913-s001.zip › supplementary materials/Figure S4.png]

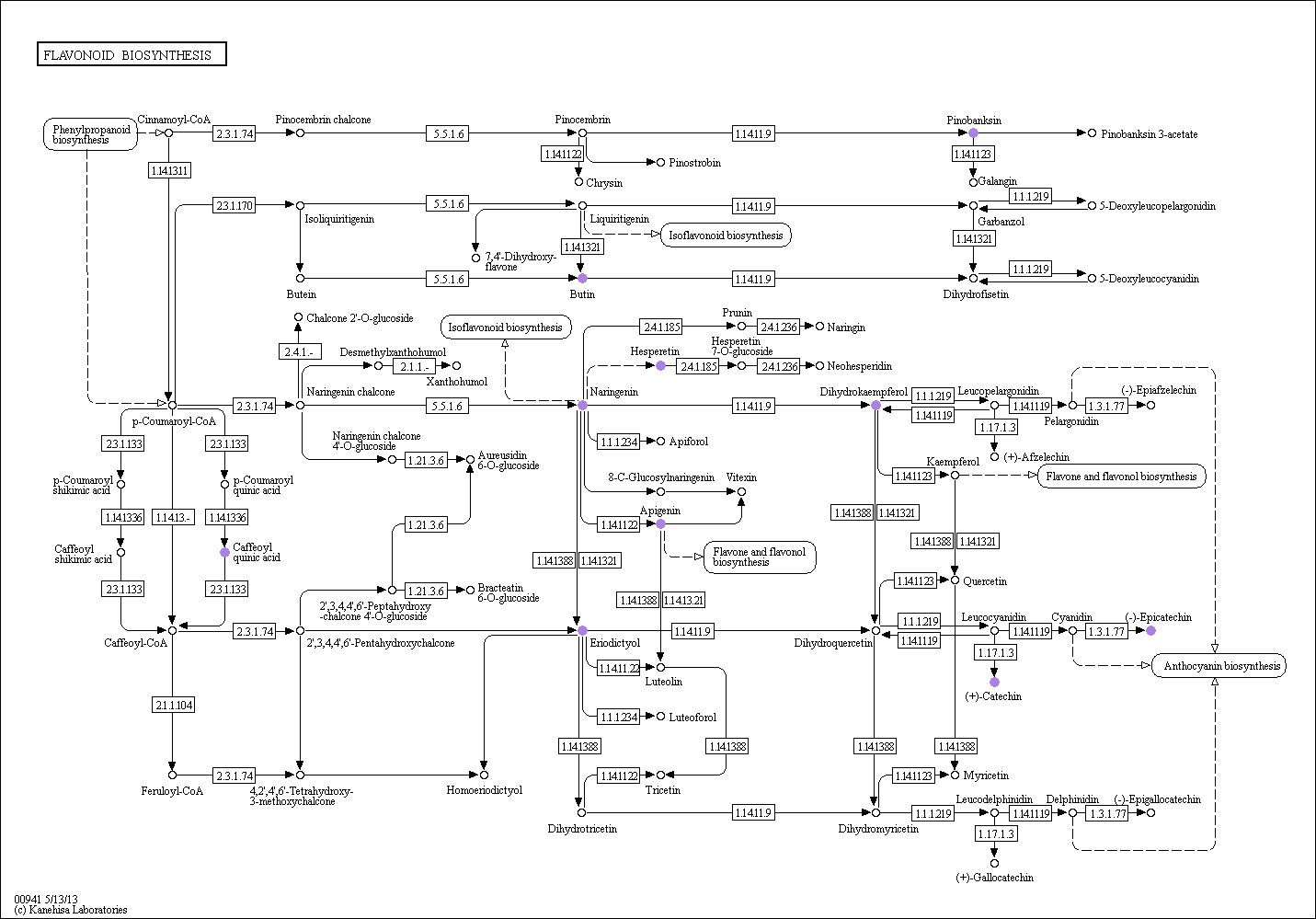

Supplement: Supplementary file 1 [file molecules-26-06913-s001.zip › supplementary materials/Figure S5.png]

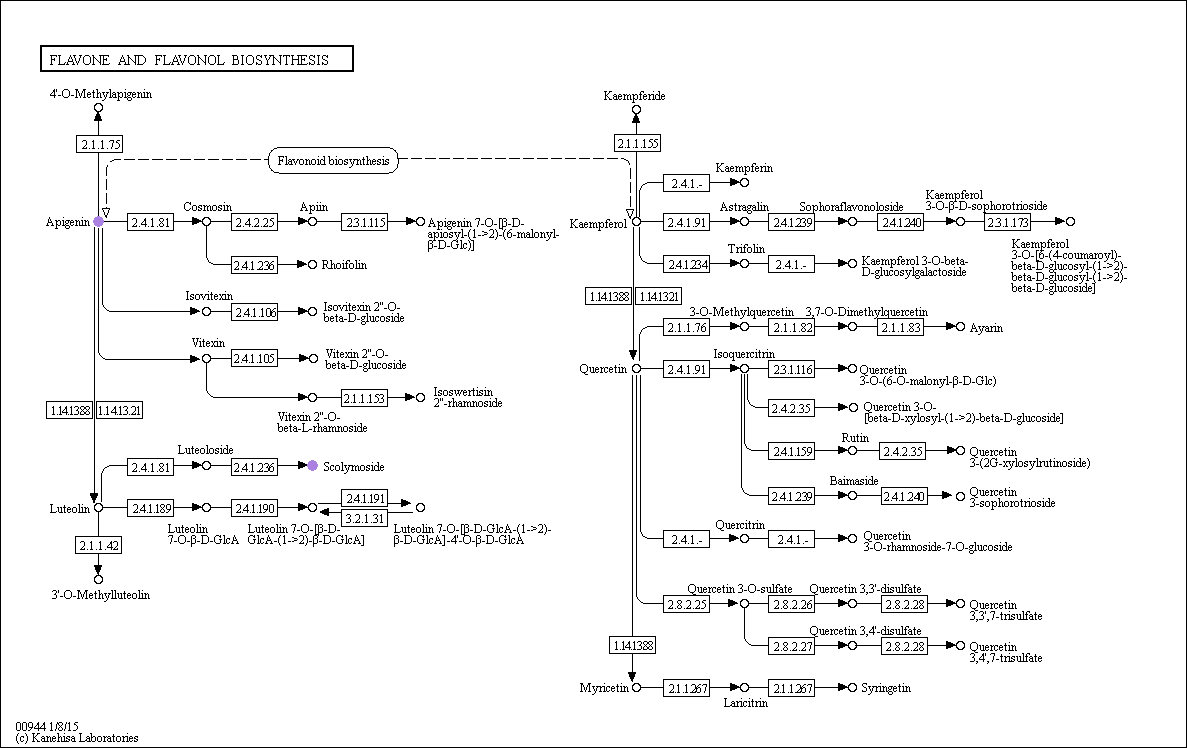

Supplement: Supplementary file 1 [file molecules-26-06913-s001.zip › supplementary materials/Figure S6.png]
